# Effects of Mechano-Sonic Vibration Therapy on Muscle Strength, Pain, and Joint Function in Elderly Patients Undergoing Total Knee and Hip Arthroplasty: A Retrospective, Case-Control Study

**DOI:** 10.3390/jpm16050253

**Published:** 2026-05-06

**Authors:** Raoul Saggini, Domiziano Tarantino, Claudia Barbato, Raffaello Pellegrino, Francesco Pegreffi, Rosa Grazia Bellomo

**Affiliations:** 1 Department of Theoretical and Applied Sciences (DiSTA), eCampus University, 22060 Novedrate, Italy; raoul.saggini@uniecampus.it (R.S.); claudiabarbato2017@gmail.com (C.B.); 2 Department of Medicine and Surgery, LUM University, 70010 Casamassima, Italy; r.pellegrino@lum.it (R.P.); bellomo@lum.it (R.G.B.); 3 Department of Medicine and Surgery, School of Medicine and Surgery, “Kore” University of Enna, 94100 Enna, Italy; francesco.pegreffi@unikore.it; 4 Recovery and Functional Rehabilitation Unit, Umberto I Hospital, Azienda Sanitaria Provinciale di Enna, 94100 Enna, Italy

**Keywords:** vibration therapy, osteoarthritis, knee arthroplasty, hip arthroplasty

## Abstract

**Background:** Early recovery after total hip (THA) and total knee arthroplasty (TKA) is often limited by pain and impaired antigravity function. Mechano-acoustic vibration therapy (VT) may enhance neuromuscular activation and analgesia, but evidence in arthroplasty is scarce. **Methods:** A total of 380 patients aged ≥65 years were retrospectively identified within 3 ± 1 days after primary unilateral total hip arthroplasty (THA) or total knee arthroplasty (TKA). All patients underwent standard inpatient physiotherapy; in the VT group, mechano-acoustic vibration therapy (ViSS^®^, 30 min/day for 5 days at 200–300 Hz) was added as an adjunct treatment, whereas the control group received standard physiotherapy alone. Pain (VAS, McGill), muscle strength (MRC), thigh circumferences, and 10 s Sit-to-Stand were assessed at baseline (T0), end of treatment (T1), and 3-day follow-up (T2). **Results:** VT produced large, early and sustained improvements in both cohorts. In THA patients, VAS decreased from 7.1 ± 1.1 to 3.8 ± 0.6 at T1 and 3.0 ± 0.7 at T2 and Sit-to-Stand repetitions increased from 3.7 ± 1.9 to 6.3 ± 1.7 at T2, with significant gains in strength and circumferences. TKA VT patients showed similar patterns. Control groups reported smaller pain reductions and no clinically relevant changes in the reported outcomes. **Conclusions:** integrating a short cycle of mechano-acoustic VT into early inpatient rehabilitation after THA or TKA significantly enhances pain relief and restoration of antigravity function compared with standard physiotherapy alone. VT represents a promising adjunct to conventional rehabilitation strategies and may contribute to optimizing postoperative recovery pathways in major joint replacement.

## 1. Introduction

Osteoarthritis (OA), the most common musculoskeletal disorder [1], is a chronic, degenerative joint disease characterized by focal articular cartilage defects, osteophyte formation, subchondral bone sclerosis, and synovial capsule thickening, ultimately leading to joint pain, deformity, and dysfunction, with a substantial impact on quality of life [2]. OA is highly prevalent and burdensome, with major personal, social, and economic consequences [3]. An estimated 240 million people worldwide have symptomatic OA [4].

First-line treatment for OA is conservative and includes both non-pharmacological and pharmacological approaches. Non-pharmacological strategies encompass physical exercise, physiotherapy and physical modalities, weight loss in overweight or obese individuals, use of walking aids or braces when indicated, patient education, and self-management. Pharmacological measures include paracetamol, non-steroidal anti-inflammatory drugs, chondroprotective agents, and intra-articular therapies with corticosteroids, hyaluronic acid, or platelet-rich plasma [5,6,7].

When conservative treatment fails, surgical procedures such as hip and knee replacement are considered for advanced OA with complete loss of articular cartilage [8,9], and are among the most successful and effective therapeutic options [10,11]. Recent data from the British National Joint Registry (NJR), which has collected patient-reported outcomes since 2009, show that 95% of patients report improvement in hip and 89% in knee pain and function 6 months after surgery [12]. A recent meta-analysis of pooled survival data derived from registry studies indicates that approximately 89% of total hip replacement and 93% of total knee replacement are still functioning at 15 years [13].

Despite advances in surgical techniques and perioperative care, many individuals continue to experience significant pain and functional limitations after joint replacement [14]. Previous studies have reported that between 14% and 36% of patients show no improvement or even deterioration 12 months after total joint replacement [15,16]. For this reason, physical therapy plays a pivotal role in the postoperative phase, aiming to reduce pain, normalize neuromuscular function, and optimize gait [17]. Achieving these goals depends on restoring appropriate muscle activation patterns, recovering functional range of motion, improving proprioception and balance, and enhancing muscular strength and endurance of the lower limbs [17,18,19].

Among physical modalities, vibration therapy (VT) has gained attention as a potentially useful rehabilitative tool to decrease postoperative pain and shorten the time required to resume activities of daily living [20,21].

VT is a low-magnitude, high-frequency physical modality that provides a safe way to deliver relevant mechanical stimuli, with the goal of improving musculoskeletal strength and physical performance in the rehabilitation setting [20,22,23,24,25,26]. Two broad categories of vibrating devices are commonly used: whole-body vibration (WBV) platforms and locally applied vibration devices, i.e., mechanical focal vibration (mFV). Both approaches rely on mechanical stimulation defined by a frequency (Hz) and an oscillation amplitude (peak-to-peak micrometer displacement), but they differ in the area of application and mode of use [27].

Several studies have shown that VT can effectively alleviate musculoskeletal pain, both when delivered via WBV [28,29] and mFV [30,31], although the precise mechanisms underlying its analgesic effects remain under investigation [20,32]. It is generally believed that stimulation of Pacinian corpuscles plays a key role, as these mechanoreceptors can modulate pain perception—either attenuating or, less commonly, exacerbating it—depending on the frequency and amplitude of the applied vibrations [33]. The pain-modulating effects of vibration are thought to be primarily mediated by activation of Aβ fibers, engagement of limbic circuits, downregulation of local TRPV1 and calcitonin expression, and the release of oxytocin [34]. By contrast, the inflammatory component of pain appears to be less influenced by vibrational stimulation, as such treatments do not seem to substantially modify cortisol levels or opioid neuropeptide release within the targeted tissues [34].

Among stimulation parameters, frequency appears to exert a stronger influence on analgesic effects than intensity, with the most effective range generally reported between 100 and 250 Hz [34]. This is consistent with previous findings on mFV, where frequencies between 100 and 300 Hz have shown the greatest efficacy in modulating both pain and muscle tone [20]. Frequencies around 100–120 Hz are considered particularly effective for promoting muscle relaxation in areas of increased tension [20,35].

Muscle fibers can be directly activated by mFV, selectively stimulating Ia and group II afferent fibers as well as Golgi tendon organs, depending on the selected application frequency, thereby improving proprioceptive responses and muscle trophism [36,37]. Regarding the sensitivity of proprioceptors to low-amplitude vibration applied to muscle tendons, Ia fibers have been shown to be the most sensitive, responding in a one-to-one fashion up to approximately 180 Hz—firing harmonically with vibrations up to about 80 Hz and then subharmonically at higher frequencies [38]. Under conditions of tonic contraction of the receptor muscle, Golgi tendon organs increase their sensitivity to vibratory stimuli [38].

Aboutorabi et al. [39] demonstrated a positive effect of mFV on balance and gait in older adults; the use of vibrating insoles combined with limited ankle and foot mFV provided reliable improvements. In addition, the mFV approach may deliver mechanical stimulation to bone and muscle–tendon structures, thereby mimicking some aspects of motion and exercise and influencing muscle function and recovery [40].

To date, only one study by Kędzierska et al. [41] has evaluated the effectiveness of VT in the early postoperative rehabilitation of post-TKR and post-THR patients. The authors reported that VT reduced pain more significantly than kinesiotherapy alone, clinically improved the absorption of post-traumatic hematomas in post-TKR patients, and, in post-THR patients treated with an anterolateral approach, significantly accelerated the recovery of normal internal rotation angles in the operated hip.

Based on these premises and considering the limited amount of research in this area, the present study aims to assess the effectiveness of mechano-acoustic VT in managing pain and its impact on muscle strength, tone, and endurance during early postoperative rehabilitation in patients who have undergone total hip arthroplasty (THA) or total knee arthroplasty (TKA).

## 2. Materials and Methods

### 2.1. Study Design and Ethical Statement

This was a retrospective comparative cohort study based on routine clinical records retrieved from the databases of the Orthopaedics Rehabilitation Department of the ICOT Institute “Marco Pasquali”, Latina, Italy. Consecutive eligible patients were included provided that they met the predefined inclusion and exclusion criteria. No balancing procedure was applied between THA and TKA cohorts. The study was based on chart review of routine clinical practice data, and no formally documented prospective allocation sequence was available for retrospective verification.

The data covered patient admissions from February 2023 to February 2024. Data processing was carried out by the leading author of the present study (R.S) and director of the above-mentioned Orthopaedics Rehabilitation Department. Subsequently, two authors of this study (R.S., D.T.) conducted further analyses to develop a customized database containing clinical information and anonymized personal data. The unique digital code assigned at the outset of this study phase ensured the maintenance of patient anonymity. The sampling method and the evaluation tools were shared between the involved researchers. The study was performed in accordance with the standard of good clinical practice and in accordance with the Helsinki Declaration of 1975. All patients included in the database provided informed consent before undergoing treatment, after which comprehensive clinical data were analyzed. The informed consent process followed these steps: (1) explanation of the treatment to the patient, (2) patient agreement, (3) completion of the consent form, (4) data verification, (5) signature, and (6) assignment of an anonymous code. The present study followed the STROBE guidelines for case-control studies [42].

### 2.2. Inclusion and Exclusion Criteria

Inclusion criteria were as follows: age ≥ 65 years; primary unilateral THA or TKA for advanced osteoarthritis with surgical indication; postoperative clinical stability allowing early mobilization; ability to stand and ambulate with walking aids; and adequate cognitive function to understand and follow instructions.

Exclusion criteria were as follows: age < 65 years; any neurological, cardiovascular or severe musculoskeletal condition that significantly limited participation in rehabilitation; any contraindication to VT, including history of active cancer, open wounds, eczema or unhealed skin lesions at the application sites, recent epileptic seizures, pregnancy, implanted electronic devices (pacemakers, neurostimulators), recent deep vein thrombosis, or severe cardiac arrhythmias; acute postoperative complications (e.g., infection, unstable prosthesis, uncontrolled pain); and inability or unwillingness to provide informed consent.

### 2.3. Treatment Protocol

Patients were identified within 3 ± 1 days after surgery, or once postoperative soft-tissue edema was considered sufficiently reduced to safely begin rehabilitation. All patients underwent the standard inpatient postoperative physiotherapy program delivered by experienced physiotherapists according to institutional protocols. In the VT group, mechano-acoustic vibration therapy was administered as an adjunct to standard physiotherapy for 30 min/day in total (15 min at 200 Hz and 15 min at 300 Hz) for 5 consecutive days. The control group received standard physiotherapy alone.

Because the present analysis was conducted retrospectively on de-identified/pseudonymized routine-care data and did not involve any study-driven intervention or patient contact, no prospective ethics registration code was generated.

For TKA patients, the standard motor rehabilitation program included the following: breathing and coordination exercises; isometric activation of quadriceps and gluteal muscles (gluteus maximus and medius); gentle patellar mobilization; active and assisted ankle dorsiflexion–plantarflexion; training in postural transitions (in-bed movements, bed–wheelchair–chair transfers, transfers to the treatment table and bathroom); trunk control and stabilization exercises; triple flexion–extension of the operated limb; limb elevation and antalgic positioning combined with manual therapy (drainage and/or decontracting massage); stretching to correct maladaptive postures; passive and active-assisted hip flexion with the knee extended (FAGE), abduction–adduction, and progressive joint recovery (passive and active-assisted) of the contralateral limb; gradual knee flexion–extension, including continuous passive motion (CPM) when available; gait training (walking with two crutches, assisted and monitored), with parallel bars used to improve weight-bearing and knee extension; and proprioceptive training.

For THA patients, the standard rehabilitation program was similar but adapted to the surgical approach. After a posterolateral approach, early rehabilitation avoided hip flexion > 60°, internal rotation and adduction. After an anterior approach, early rehabilitation avoided bridging (supine bridge position), prone positioning, excessive hip extension, and excessive external rotation or large combined movements. Gait training started with a walker (with underarm support when needed), always under physiotherapist supervision.

All patients had access to as-needed analgesia and could receive paracetamol 1000 mg up to three times per day, or ketorolac 30 mg intramuscularly, up to once per day.

### 2.4. VT Device

**Figure 1:**
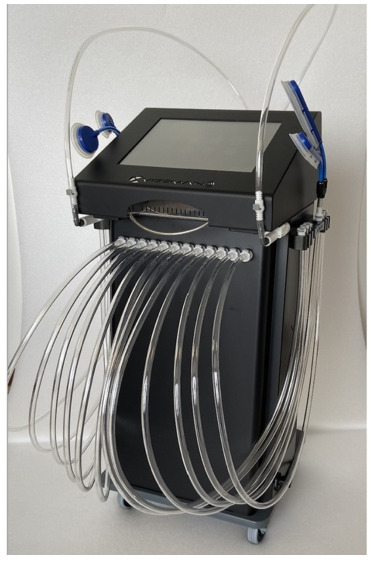
ViSS^®^ (Vibration Sound System^®^) device.

VT was delivered using the ViSS^®^ (Vibration Sound System^®^) device (Vissman Europe s.r.l., Rome, Italy) (Figure 1), consisting of a blower with an airflow capacity of 35 m^3^/h and a peak pressure of 250 mBar, and a flow modulator that generates alternating positive/negative pressure waves, producing mechano-acoustic vibrations with adjustable pressure up to 630 mBar.

The device can generate frequencies up to 980 Hz; in this protocol, frequencies were limited to ≤300 Hz in accordance with safety recommendations and standard operating procedures.

**Figure 2:**
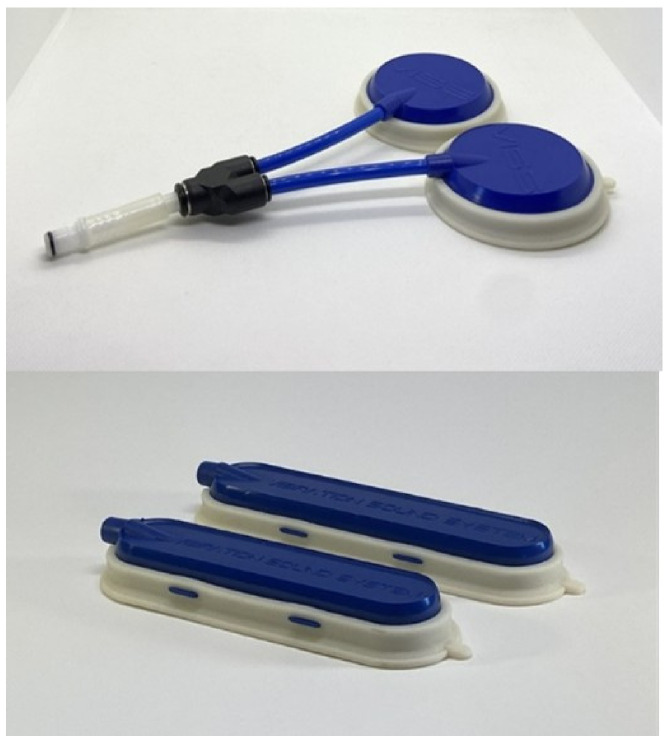
Types of transducers.

Mechano-acoustic vibration was delivered through transducers fitted with a “gull-wing” sound-damping pad designed to adhere firmly to the skin, reduce air leakage and adapt to individual body contours. Transducers of various shapes and sizes were used to ensure optimal contact (Figure 2).

**Figure 3:**
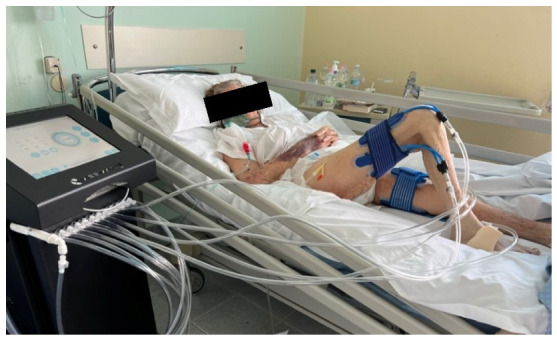
Transducer applications on specific sites of the lower limbs.

Some models included a membrane positioned between the polymer and the cushioning layer to further limit leakage and provide more uniform transmission of vibrations across the entire contact surface. Transducers were applied to the gluteus maximus, quadriceps femoris, hamstrings, and tibialis posterior muscles (Figure 3).

These muscles were selected due to their antigravity role and importance in standing and gait.

### 2.5. Outcome Measures and Assessment Schedule

Each patient was assessed at three time points: T0 (Baseline), before the first treatment session; T1 (End of treatment), on day five at completion of the treatment cycle; and T2 (Follow-up), three days after treatment ended.

The following outcome measures were collected: pain intensity using the VAS (Visual Analogue Scale, 0–10) and the McGill Pain Questionnaire (15 descriptors: 11 sensory and 4 affective, each scored 0–3); muscle strength using the Medical Research Council (MRC) scale (0–5), based on observation of active movement (grades 0–3) and manual muscle testing (grades 4–5); muscle trophism, quantified by thigh circumference at the root of the thigh (CM ROOT) and 10 cm above the superior border of the patella (CM ABOVE PATELLA); and muscle endurance/functional performance using the 10 s Sit-to-Stand test (Sit-to-Stand 10 s), adapted for this population with a four-wheeled walker to ensure safety.

Primary outcomes were pain reduction (VAS and McGill) and functional performance (Sit-to-Stand). Muscle strength (MRC) and circumferences (CM ROOT and CM ABOVE PATELLA) were considered secondary outcomes. The selected outcomes reflect the measures consistently available in the retrospective early postoperative rehabilitation records.

Available medical records were also reviewed for reported adverse events, pain worsening, postoperative complications, or treatment-related intolerance during the observation period.

### 2.6. Statistical Analysis

Data were analyzed using repeated-measures ANOVA to assess changes over time (T0, T1, T2) within each group (VT vs. control) for THA and TKA patients separately. For each variable, F statistics, *p* values, and partial eta squared (φp^2^) were reported. Assumption of sphericity was checked with Mauchly’s test; when violated, Greenhouse–Geisser correction was applied.

Post-hoc comparisons between time points were conducted with Bonferroni correction where appropriate. To better characterize the pattern of change, trend analyses were carried out, and regression coefficients (R^2^) were calculated for the trajectories of pain and functional variables in treatment vs. control groups. The level of statistical significance was set at *p* < 0.05 for all analyses.

Because the retrospective dataset available for manuscript revision did not preserve patient-level longitudinal values for post hoc reconstruction, formal inferential between-group testing of individual change scores could not be reliably performed. Therefore, additional between-group change-score comparisons are presented descriptively, using mean differences from baseline to end of treatment (ΔT1−T0) and from baseline to follow-up (ΔT2−T0).

## 3. Results

A total of 380 patients were included. The THA cohort comprised 200 patients with a mean age of 73 years (range 65–78): 100 women (50 VT, 50 control) and 100 men (50 VT, 50 control), yielding 100 patients in the VT group and 100 in the control group (Table 1). The TKA cohort comprised 180 patients with a mean age of 72 years (range 65–79): 100 women (50 assigned to VT and 50 to control) and 80 men (40 assigned to VT and 40 to control), resulting in 90 patients in the VT group and 90 in the control group.

The numerical difference between THA and TKA cases reflects the composition of the consecutive eligible records available during the study period.

### 3.1. THA Cohort

In THA patients treated with VT, repeated-measures ANOVA showed statistically significant improvements with large effect sizes in all outcomes between baseline and end of treatment, and between baseline and follow-up. Pain intensity (VAS and McGill) decreased markedly at T1 and declined further at T2. Muscle strength (MRC) increased significantly after treatment. Thigh circumference measures (CM ROOT and CM ABOVE PATELLA) also changed over time; however, these short-term variations should be interpreted cautiously in the early postoperative setting, as they are more likely to reflect a combination of edema redistribution/resolution, local tissue status, and measurement variability rather than true structural muscle hypertrophy. Circumference values showed a slight reduction from T1 to T2 but remained above baseline.

**Figure 4:**
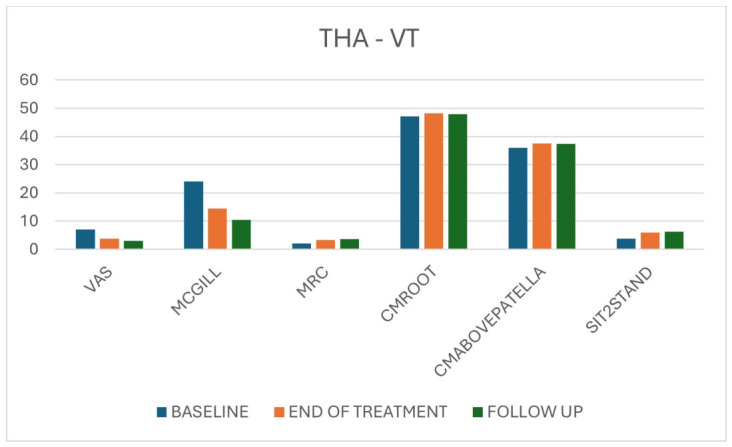
Overall results for THA patients in the VT group across the three time points (T0, T1, T2) for pain, strength, circumference and Sit-to-Stand.

Taken together, these findings indicate an early and sustained benefit of VT on pain, strength, muscle trophism and functional performance (Table 2, Figure 4).

**Figure 5:**
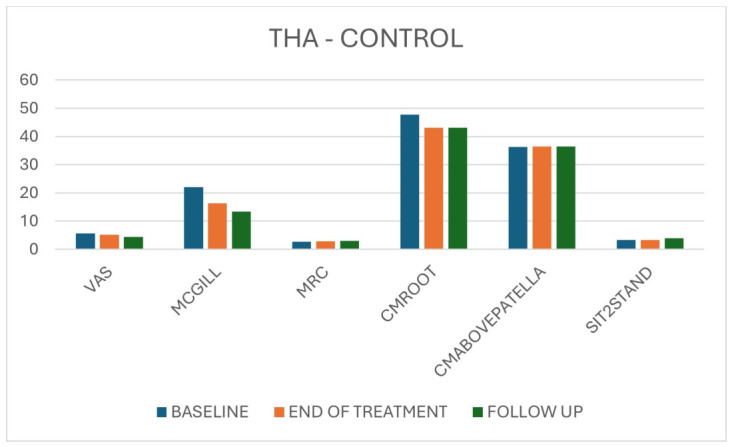
Overall results for THA patients in the control group across the three time points (T0, T1, T2) for pain, strength, circumference and Sit-to-Stand.

In the THA control group, strength, muscle circumferences and functional performance did not change significantly over time, apart from a moderate reduction in VAS and McGill scores and a significant Sit-to-Stand improvement only between baseline and follow-up. Effect sizes for pain reduction were smaller than in the VT group, and functional gains were later in onset and less marked (Table 3, Figure 5).

**Figure 6:**
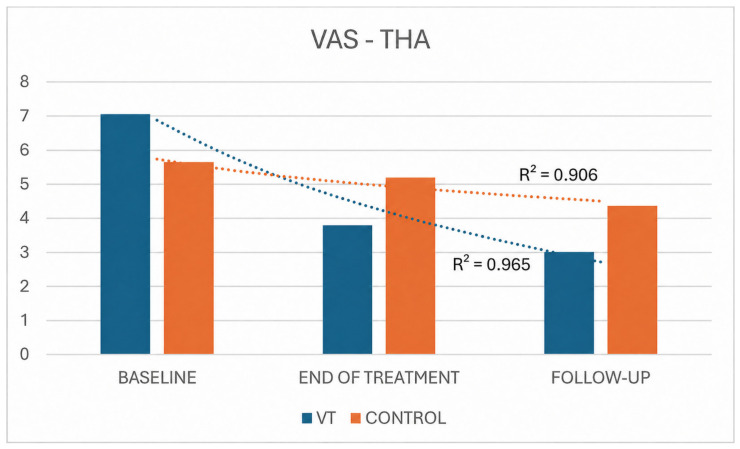
VAS comparison between THA VT and control groups across time.

**Figure 7:**
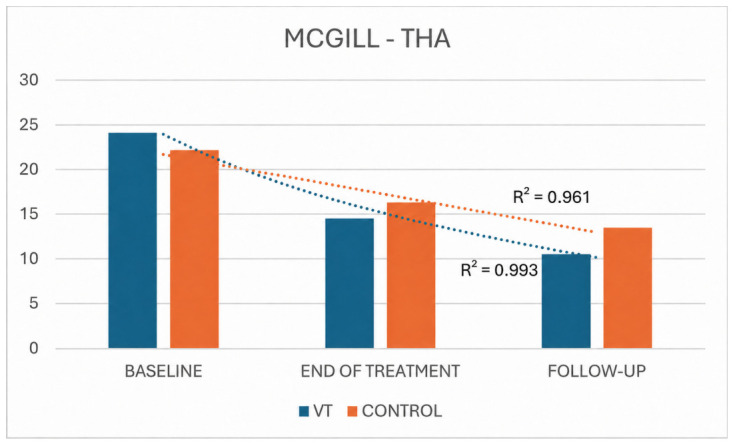
McGill Pain Questionnaire scores in THA VT and control groups across time.

For both pain outcomes (VAS and McGill), statistically significant changes over time were observed in both THA groups. Overall, the VT group exhibited a more pronounced decline in symptoms than the control group, as reflected by higher coefficients of determination for both VAS (R^2^ = 0.965 vs. 0.906 for treatment and control, respectively) and McGill scores (R^2^ = 0.993 vs. 0.961, respectively) (Figure 6 and Figure 7).

To improve between-group interpretability, descriptive mean change scores from baseline were also calculated. For VAS, mean change was −3.26 at T1 and −4.05 at T2 in the VT group, compared with −0.46 and −1.28, respectively, in the control group. For McGill scores, mean change was −9.58 at T1 and −13.58 at T2 in the VT group, compared with −5.82 and −8.64, respectively, in the control group.

**Figure 8:**
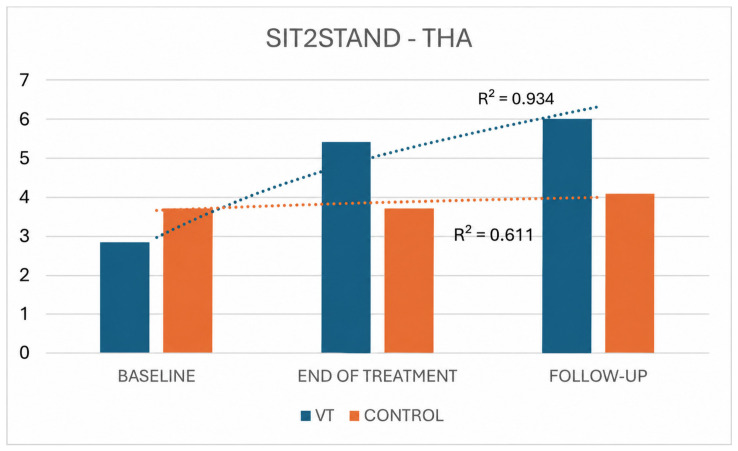
Sit-to-Stand performance trends in THA VT and control groups.

Regarding the 10 s Sit-to-Stand test in the THA cohort, repeated-measures ANOVA showed a clear and progressive functional gain in the VT group, whereas the control group exhibited only a delayed and modest improvement. In VT-treated patients, the mean number of Sit-to-Stand repetitions increased from 3.74 ± 1.88 at baseline to 5.95 ± 1.65 at the end of treatment and 6.26 ± 1.69 at follow-up (F = 73.663, *p* < 0.001, ηp^2^ = 0.89), corresponding to a net gain of +2.21 repetitions from baseline to treatment end and +2.52 from baseline to follow-up. By contrast, control patients showed virtually unchanged performance between baseline and the end of treatment (3.27 ± 1.10 vs. 3.27 ± 0.78), with a statistically significant improvement emerging only at follow-up (4.00 ± 0.89; F = 7.110, *p* = 0.014, ηp^2^ = 0.61), for an overall gain of +0.73 repetitions. The improvement trend (R^2^) was significantly higher in the VT group than in the control group (R^2^ = 0.934 and R^2^ = 0.611, respectively) (Figure 8).

### 3.2. TKA Cohort

**Figure 9:**
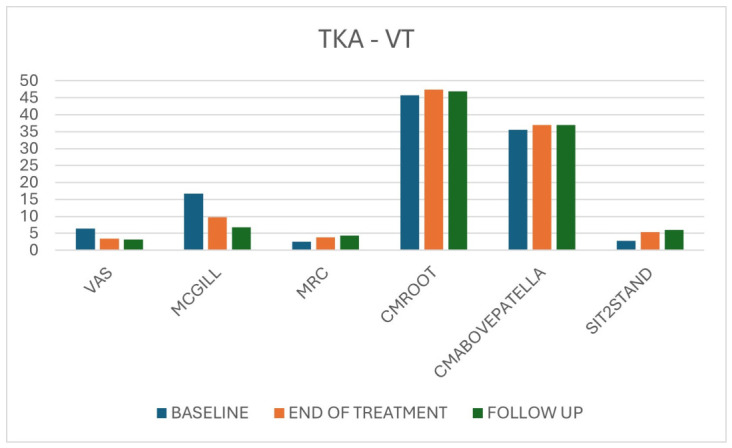
Overall results for TKA patients in the VT group across the three-time points.

In TKA patients treated with VT, repeated-measures ANOVA again demonstrated statistically significant and clinically relevant improvements for all variables between T0 and T1 and between T0 and T2. Pain scores (VAS and McGill) decreased significantly with high effect sizes; strength (MRC), thigh circumferences and Sit-to-Stand performance all improved progressively across the three time points. Improvements were already evident at T1 and were maintained or further enhanced at T2 (Table 4; Figure 9).

**Figure 10:**
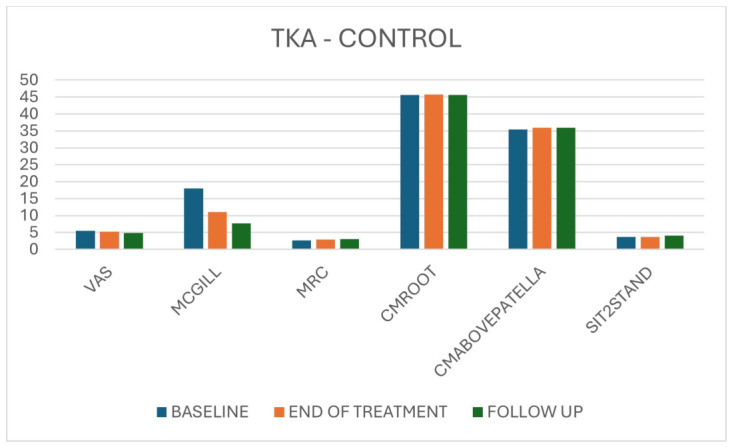
Overall results for TKA patients in the control group across the three time points.

In TKA controls, pain scores decreased significantly over time, but effect sizes were smaller than in the VT group. Strength, limb circumferences and Sit-to-Stand performance did not show significant or clinically meaningful changes, except for minor fluctuations without a clear trend. In contrast, graphical comparisons confirmed that both the magnitude and slope of pain reduction were greater in the VT groups, and that functional gains in Sit-to-Stand were earlier and more pronounced with VT (Table 5, Figure 10).

**Figure 11:**
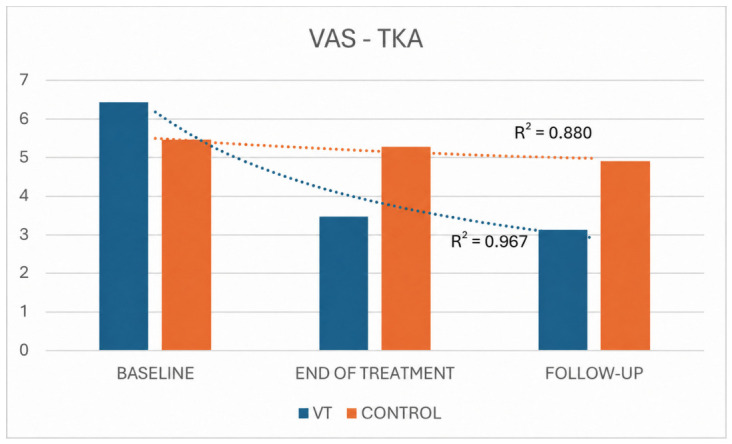
VAS comparison between TKA VT and control groups across time.

**Figure 12:**
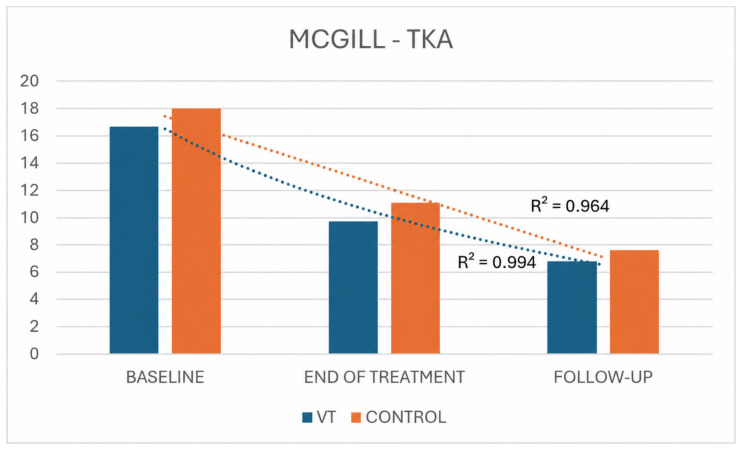
McGill Pain Questionnaire scores in TKA VT and control groups across time.

In the TKA group, as in the THA group, both pain-related variables (VAS and McGill) showed statistically significant improvements in the VT and control groups. However, Figure 11 and Figure 12 clearly illustrate that the treatment group exhibited a steeper and more consistent decline in symptoms, as reflected by higher coefficients of determination (VAS: R^2^ = 0.967 vs. 0.880 for treatment and control; McGill: R^2^ = 0.994 vs. 0.964).

**Figure 13:**
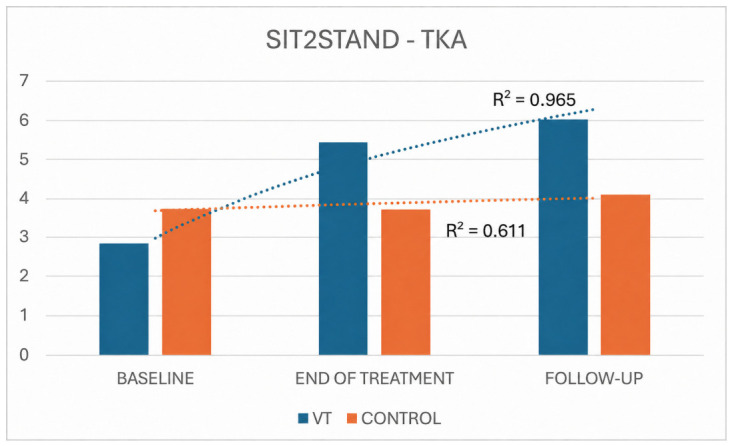
Sit-to-Stand performance trends in TKA VT and control groups.

Regarding the 10 s Sit-to-Stand test in the TKA cohort, repeated-measures ANOVA likewise demonstrated a clear and progressive functional gain in the VT group, whereas the control group showed only minimal, non-significant change over time. In VT-treated patients, the mean number of Sit-to-Stand repetitions increased from 2.84 ± 0.77 at baseline to 5.42 ± 1.43 at the end of treatment and 6.05 ± 1.72 at follow-up (F = 85.037, *p* < 0.001, ηp^2^ = 0.91), corresponding to a net gain of +2.58 repetitions from baseline to treatment end and +3.21 from baseline to follow-up. By contrast, control patients again showed virtually unchanged performance between baseline and the end of treatment (3.73 ± 1.01 vs. 3.73 ± 0.79), with only a small, non-significant increase to 4.09 ± 0.70 at follow-up (F = 3.200, *p* = 0.089, ηp^2^ = 0.42), for an overall gain of +0.36 repetitions. The improvement trend (R^2^) was significantly higher in the VT group than in the control group (R^2^ = 0.965 and R^2^ = 0.611, respectively) (Figure 13).

### 3.3. Descriptive Between-Group Change-Score Comparison

To improve the interpretability of treatment effects, mean change scores from baseline to end of treatment (ΔT1−T0) and from baseline to follow-up (ΔT2−T0) were also calculated descriptively for the VT and control groups. These comparisons were based on group mean values at each time point and are presented as descriptive estimates of the magnitude of improvement over time rather than as formal inferential between-group tests (Table 6).

For descriptive purposes, mean change scores from baseline were also calculated for pain outcomes in the VT and control groups. In the THA cohort, VAS mean change was −3.26 at T1 and −4.05 at T2 in the VT group, compared with −0.46 and −1.28, respectively, in the control group. McGill mean change was −9.58 at T1 and −13.58 at T2 in the VT group, compared with −5.82 and −8.64, respectively, in the control group. In the TKA cohort, VAS mean change was −2.95 at T1 and −3.31 at T2 in the VT group, compared with −0.18 and −0.54, respectively, in the control group. McGill mean change was −6.94 at T1 and −9.89 at T2 in the VT group, compared with −6.91 and −10.36, respectively, in the control group.

### 3.4. Safety and Tolerability

No adverse events, treatment-related complications, or documented pain worsening attributable to VT were recorded in the available medical charts during the observation period in either group.

## 4. Discussion

This study shows that integrating mechano-acoustic VT into the very early phase of rehabilitation after THA and TKA produces clinically relevant improvements in pain, muscle performance and functional mobility compared with standard rehabilitation alone. Across both cohorts, VT-treated patients exhibited a steeper and more regular decline in pain scores and a markedly greater improvement in Sit-to-Stand performance than controls, with large effect sizes and high coefficients of determination for the change over time. These findings indicate that mechano-acoustic VT not only reduces pain but also accelerates the recovery of antigravity function, which is a key prerequisite for safe transfers, gait re-training and early autonomy after major joint replacement. The mechanisms underlying these effects are likely multifactorial. From a neurophysiological perspective, mechano-acoustic vibration at frequencies between 100 and 300 Hz can selectively activate Ia afferents and other mechanoreceptors, modulate the tonic vibration reflex, and induce plastic changes in spinal and supraspinal circuits, with consequent improvements in motor unit recruitment and coordination [22]. At the same time, the stimulation of Pacinian corpuscles and Aβ fibers may engage segmental and suprasegmental pain control mechanisms, altering nociceptive transmission and the affective component of pain without substantially affecting the inflammatory milieu [20,22]. This dual action on pain and motor control provides a plausible framework for the combined reduction in pain intensity and the enhancement of Sit-to-Stand performance observed in our THA and TKA cohorts [41].

Compared with the only previous study specifically investigating vibroacoustic therapy in post-THR and post-TKR patients, by Kędzierska et al. [41], our results both confirm and extend the available evidence. Kędzierska et al. evaluated 60 patients (post-TKR and post-THR) randomized to standard kinesiotherapy with or without early vibroacoustic therapy delivered during the first four postoperative days. They found that VT significantly enhanced pain relief in post-TKR patients compared with kinesiotherapy alone, while the reduction in limb circumference and groin oedema, as well as improvements in knee and hip range of motion, were either not associated with vibroacoustic therapy or occurred similarly in both groups. In the THR cohort, vibroacoustic therapy modestly accelerated the recovery of internal rotation in patients treated with an anterolateral approach but did not translate into broader functional gains. Overall, that study supported a short-term analgesic effect of vibroacoustic therapy yet suggested only limited additional impact on oedema resolution and active joint mobility.

Our data confirm the analgesic potential of vibration-based interventions after major joint replacement but show a broader spectrum of benefit, particularly on functional performance and muscle-related outcomes. In both THA and TKA cohorts, pain reduction in the VT groups was large and progressive, with VAS and McGill scores showing very high effect sizes and steeper regression slopes than in controls. However, in contrast to Kędzierska et al., we also documented substantial gains in Sit-to-Stand performance and in muscle strength and trophism in the VT groups, while controls showed almost flat trajectories for Sit-to-Stand and only limited or absent change in MRC and thigh circumferences. This suggests that, when integrated into a task-oriented rehabilitation pathway and targeted to antigravity muscle chains, mechano-acoustic vibration may facilitate neuromuscular recovery beyond analgesia alone.

Several methodological differences may explain the discrepancies between the two studies. First, the timing and “dose” of vibration differ markedly. Kędzierska et al. delivered vibroacoustic therapy as four short sessions confined to the first postoperative days, in a phase dominated by surgical trauma, inflammation and immobilization [41]. In our protocol, mechano-acoustic vibration was administered over a structured five-day cycle with systematic integration into early mobilization and strengthening exercises, and outcomes were assessed not only at the end of the treatment cycle but also three days later, when patients had already engaged in more active rehabilitation. Second, the technical characteristics of the devices and the target tissues differ. Kędzierska et al. used a vibroacoustic “phoning” approach focused on the operated region, whereas we used a ViSS^®^ system delivering mFV (≤300 Hz) through transducers applied to gluteus maximus, quadriceps, hamstrings and tibialis posterior, i.e., the main antigravity muscles involved in Sit-to-Stand and gait. Third, the choice of outcome measures diverges: Kędzierska et al. primarily assessed pain, oedema, limb circumference and joint range of motion, all important but not directly reflective of task-specific function; by contrast, we selected the 10 s Sit-to-Stand test as a primary functional endpoint, alongside pain, strength and trophism, thereby capturing changes in neuromuscular performance that may not be evident from joint ROM alone.

Beyond this direct comparison, our findings are coherent with data from other clinical applications of mechano-acoustic and mFV. In chronic stroke, repeated local high-frequency vibration with the same ViSS^®^ technology (300 Hz, 12 sessions over four weeks) has been shown to significantly decrease muscle tone and pain and to improve grip strength and functional scores, suggesting an enhancement of corticospinal excitability and motor unit recruitment [43]. In knee osteoarthritis, the combination of intra-articular oxygen–ozone therapy with mFV applied to quadriceps and related muscles resulted in greater improvements in pain, KOOS subscales, quadriceps strength and knee ROM than infiltrative therapy alone, again using a ViSS-based protocol (300 Hz, multiple sessions) [44]. More recently, mFV have been reported to improve pain, posture and functional outcomes helping to improve mobility, preserve functional autonomy, and enhance quality of life in different post-surgical [20], chronic musculoskeletal such as OA [45,46], and neurological populations [24,35,47,48,49], reinforcing the concept that the clinical effect depends on adequate frequency, cumulative exposure and accurate muscle targeting [20,25]. Within this broader context, the present study is, to our knowledge, the first to show that a short, intensive cycle of mechano-acoustic VT integrated into early rehabilitation can influence both pain trajectories and an objective, functionally meaningful task such as repeated Sit-to-Stand in patients after THA and TKA.

### Strengths and Limitations

Strengths of this study include the inclusion of two major arthroplasty populations (THA and TKA) with parallel control groups, the use of a standardized mechano-acoustic vibration protocol applied to functionally relevant muscle groups, and the adoption of clinically grounded endpoints that encompass pain, strength, trophism and a task-specific functional test. The repeated-measures design with assessments at baseline, end of treatment and short-term follow-up allows characterization of the temporal pattern of change and suggests that benefits extend beyond the immediate stimulation period. Furthermore, the protocol is pragmatic and compatible with routine inpatient rehabilitation, which supports its potential translational value.

Several limitations should be acknowledged. First, although the sample size is comparable with that of other early postoperative rehabilitation studies, it may still limit statistical power for subgroup analyses and for the identification of predictors of response.

Second, the retrospective comparative design introduces important methodological constraints. Because no prospectively documented allocation sequence was available for retrospective verification, both selection bias and allocation bias cannot be excluded.

In addition, the numerical imbalance between THA and TKA cases reflects the composition of the consecutive eligible records available during the study period rather than a prospectively balanced sampling strategy, and cohort distribution may therefore have been influenced by the characteristics of the available dataset.

Third, the absence of blinding represents a further limitation. Given the retrospective real-world design and the nature of the intervention, neither participant nor assessor blinding was feasible or documentable. Consequently, expectation effects, additional attention, and performance-related bias may have influenced subjective outcomes, such as pain ratings, and, to a lesser extent, functional performance measures. Accordingly, the observed short-term improvements, particularly in patient-reported pain outcomes, should be interpreted with caution.

Fourth, follow-up was limited to the immediate post-treatment period. Therefore, the present study does not allow conclusions regarding the mid- or long-term persistence of pain reduction, functional recovery, or fall-related outcomes after arthroplasty. Future prospective studies should include longer follow-up intervals and clinically relevant endpoints, such as sustained functional improvement and fall risk.

In addition, several potentially relevant baseline variables, including BMI, individual comorbidity burden, and preoperative functional status, were not consistently available in the retrospective clinical records and therefore could not be included in the analysis. Residual confounding cannot therefore be excluded.

Similarly, although a rescue analgesic protocol was available to all patients, actual individual analgesic consumption was not consistently recorded and could not be reliably reconstructed or compared between groups. Moreover, not all patients required analgesics and, among those who did, drug type, dosage, frequency, and duration varied across individuals. Analgesic use may therefore have influenced both pain scores and functional performance, representing an additional source of confounding.

Another limitation is the lack of patient-level longitudinal data for post hoc reconstruction of formal between-group change-score analyses. As a result, comparisons of ΔT1−T0 and ΔT2−T0 are reported descriptively and should not be interpreted as inferential tests of treatment effect.

The study is also limited by the absence of validated arthroplasty-specific functional outcome measures, such as WOMAC, KOOS, or other standardized joint-specific postoperative scales. Because these measures were not routinely collected in the retrospective inpatient rehabilitation database, they could not be reconstructed post hoc. This limits both the clinical interpretation of functional recovery and the comparability of our findings with the broader arthroplasty literature.

Finally, we did not include objective biomechanical or neurophysiological measures, such as gait analysis or electromyography, which might have helped to clarify the relative contribution of neuromuscular and analgesic mechanisms.

As with most single-center studies, the external validity of these findings to other settings, surgical techniques, and rehabilitation models remains uncertain and should be tested in future multicenter prospective trials.

## 5. Conclusions

This study demonstrates that mechano-acoustic VT, when integrated into postoperative rehabilitation, enhances pain reduction and functional recovery after THA and TKA. Patients treated with VT showed earlier and more pronounced improvements in Sit-to-Stand performance and reported lower pain scores compared to those receiving standard rehabilitation alone. These results extend previous evidence by showing that the therapeutic benefit of VT is strongly dependent on cumulative exposure and structured integration within rehabilitation protocols. Mechano-acoustic VT thus represents a promising adjunct to conventional rehabilitation strategies and may contribute to optimizing postoperative recovery pathways in major joint replacement. Further research is warranted to refine treatment parameters and confirm long-term clinical benefits.

## Figures and Tables

**Table 1:** Demographic and basic clinical characteristics of the study population undergoing THA or TKA. Age ranges are reported as in the original dataset. Percentages are calculated within each column.

Variable	THA (n = 200)	TKA (n = 180)	Total (N = 380)
**Age, years**			
Mean (range)	73 (65–78)	72 (65–79)	72.5 (65–79) *
**Sex, n (%)**			
Female	100 (50.0%)	100 (55.6%)	200 (52.6%)
Male	100 (50.0%)	80 (44.4%)	180 (47.4%)
**Treatment group, n (%)**			
VT group	100 (50.0%)	90 (50.0%)	190 (50.0%)
Control group	100 (50.0%)	90 (50.0%)	190 (50.0%)
**Perioperative characteristics**			
THA surgical approach	Minimally invasive anterior approach	—	—
Anesthesia	Spinal (subarachnoid) anesthesia	Spinal (subarachnoid) anesthesia	Spinal (subarachnoid) anesthesia
Operative time	Approximately 60 min	Approximately 60 min	Approximately 60 min
**Postoperative rescue analgesia**			
Paracetamol 1000 mg up to three times daily or ketorolac 30 mg intramuscularly up to once daily, as needed	Yes	Yes	Yes

* Total mean age reported as approximate midpoint based on THA and TKA mean ages.

**Table 2:** Repeated-measures ANOVA for patients with THA in the VT group.

Measure	Time Point	M	SD	F	*p*	φp^2^	Post-Hoc
**VAS**	Baseline (1)	7.05	1.13	179.515	<0.001	0.94	1 > 2 > 3
	End treatment (2)	3.79	0.63				
	Follow-up (3)	3.00	0.67				
**McGill**	Baseline	24.11	7.56	53.418	<0.001	0.86	1 > 2 > 3
	End treatment	14.53	5.05				
	Follow-up	10.53	5.55				
**MRC**	Baseline	2.11	0.57	117.111	<0.001	0.93	1 < 2; 1 < 3
	End treatment	3.37	0.68				
	Follow-up	3.58	0.51				
**CM ROOT**	Baseline	47.16	3.92	12.750	<0.001	0.60	1 < 2; 1 < 3; 2 > 3
	End treatment	48.26	3.68				
	Follow-up	47.95	3.76				
**CM ABOVE PATELLA**	Baseline	36.00	4.45	33.670	<0.001	0.86	1 < 2; 1 < 3; 2 > 3
	End treatment	37.54	4.21				
	Follow-up	37.38	4.25				
**Sit-to-Stand**	Baseline	3.74	1.88	73.663	<0.001	0.89	1 < 2; 2 < 3
	End treatment	5.95	1.65				
	Follow-up	6.26	1.69				

**Legend:** M = mean; SD = standard deviation; φp^2^ = partial eta squared; VAS = Visual Analogue Scale; CM = circumference; Sit-to-Stand = 10 s Sit-to-Stand test.

**Table 3:** Repeated-measures ANOVA for patients with THA in the control group.

Measure	Time Point	M	SD	F	*p*	φp^2^	Post-Hoc
**VAS**	Baseline (1)	5.64	1.21	8.062	0.010	0.64	1 > 3; 2 > 3
	End treatment (2)	5.18	1.17				
	Follow-up (3)	4.36	1.63				
**McGill**	Baseline	22.09	7.98	7.313	0.013	0.62	1 > 2 > 3
	End treatment	16.27	7.06				
	Follow-up	13.45	7.61				
**MRC**	Baseline	2.73	0.47	2.571	0.131	0.36	–
	End treatment	2.91	0.30				
	Follow-up	3.09	0.30				
**CM ROOT**	Baseline	47.73	4.52	1.000	0.341	0.09	–
	End treatment	43.18	13.96				
	Follow-up	43.18	13.96				
**CM ABOVE PATELLA**	Baseline	36.33	4.12	1.000	0.347	0.11	–
	End treatment	36.44	4.09				
	Follow-up	36.44	4.09				
**Sit-to-Stand**	Baseline	3.27	1.10	7.110	0.014	0.61	2 < 3
	End treatment	3.27	0.78				
	Follow-up	4.00	0.89				

**Table 4:** Repeated-measures ANOVA for patients with TKA in the VT group.

Measure	Time Point	M	SD	F	*p*	φp^2^	Post-Hoc
**VAS**	Baseline (1)	6.42	1.21	292.179	<0.001	0.97	1 > 2; 1 > 3
	End treatment (2)	3.47	1.12				
	Follow-up (3)	3.11	0.94				
**McGill**	Baseline	16.68	6.37	174.103	<0.001	0.95	1 > 2 > 3
	End treatment	9.74	6.06				
	Follow-up	6.79	3.14				
**MRC**	Baseline	2.58	0.51	161.075	<0.001	0.95	1 < 2 < 3
	End treatment	3.79	0.42				
	Follow-up	4.32	0.48				
**CM ROOT**	Baseline	45.75	4.65	53.000	<0.001	0.95	1 < 2; 1 < 3
	End treatment	47.38	4.63				
	Follow-up	46.88	4.61				
**CM ABOVE PATELLA**	Baseline	35.58	2.28	88.000	<0.001	0.89	1 < 2; 1 < 3
	End treatment	36.92	2.11				
	Follow-up	36.92	2.11				
**Sit-to-Stand**	Baseline	2.84	0.77	85.037	<0.001	0.91	1 < 2 < 3
	End treatment	5.42	1.43				
	Follow-up	6.05	1.72				

**Legend**: VAS = Visual Analogue Scale; M = mean; SD = standard deviation; φp^2^ = partial eta squared.

**Table 5:** Repeated-measures ANOVA for patients with TKA in the control group.

Measure	Time Point	M	SD	F	*p*	φp^2^	Post-Hoc
**VAS**	Baseline (1)	5.45	1.13	5.40	0.029	0.55	1 > 3
	End treatment (2)	5.27	1.01				
	Follow-up (3)	4.91	1.04				
**McGill**	Baseline	18.00	7.16	71.27	<0.001	0.94	1 > 2 > 3
	End treatment	11.09	6.54				
	Follow-up	7.64	3.61				
**MRC**	Baseline	2.64	0.67	2.189	0.168	0.33	–
	End treatment	2.91	0.30				
	Follow-up	3.09	0.54				
**CM ROOT**	Baseline	45.55	4.63	1.000	0.341	0.09	–
	End treatment	45.73	4.47				
	Follow-up	45.64	4.55				
**CM ABOVE PATELLA**	Baseline	35.56	3.00	4.000	0.081	0.33	–
	End treatment	35.89	3.29				
	Follow-up	35.89	3.29				
**Sit-to-Stand**	Baseline	3.73	1.01	3.200	0.089	0.42	–
	End treatment	3.73	0.79				
	Follow-up	4.09	0.70				

**Legend:** VAS = Visual Analogue Scale; M = mean; SD = standard deviation; φp^2^ = partial eta squared.

**Table 6:** Descriptive between-group change scores from baseline for pain outcomes.

Cohort	Outcome	VT T0	VT T1	VT T2	Control T0	Control T1	Control T2	VT ΔT1−T0	Control ΔT1−T0	VT ΔT2−T0	Control ΔT2−T0
THA	VAS	7.05	3.79	3.00	5.64	5.18	4.36	−3.26	−0.46	−4.05	−1.28
THA	McGill	24.11	14.53	10.53	22.09	16.27	13.45	−9.58	−5.82	−13.58	−8.64
TKA	VAS	6.42	3.47	3.11	5.45	5.27	4.91	−2.95	−0.18	−3.31	−0.54
TKA	McGill	16.68	9.74	6.79	18.00	11.09	7.64	−6.94	−6.91	−9.89	−10.36

Descriptive change scores were calculated as mean differences from baseline to end of treatment (ΔT1−T0) and from baseline to follow-up (ΔT2−T0).

## Data Availability

The data associated with the paper are not publicly available but are available from the corresponding author on reasonable request. Data are located in controlled data storage at the ICOT Institute “Marco Pasquali” of Latina, Italy.
